# Host Blood RNA Transcript and Protein Signatures for Sputum-Independent Diagnostics of Tuberculosis in Adults

**DOI:** 10.3389/fimmu.2020.626049

**Published:** 2021-02-04

**Authors:** Dhanasekaran Sivakumaran, Christian Ritz, John Espen Gjøen, Mario Vaz, Sumithra Selvam, Tom H. M. Ottenhoff, Timothy Mark Doherty, Synne Jenum, Harleen M. S. Grewal

**Affiliations:** ^1^ Department of Clinical Science, Faculty of Medicine, University of Bergen, Bergen, Norway; ^2^ Department of Microbiology, Haukeland University Hospital, University of Bergen, Bergen, Norway; ^3^ Department of Nutrition, Exercise and Sports, University of Copenhagen, Copenhagen, Denmark; ^4^ Department of Paediatrics, Haukeland University Hospital, Bergen, Norway; ^5^ Department of Physiology, St. John’s Medical College and Division of Health and Humanities, St. John’s Research Institute, Bangalore, India; ^6^ Division of Infectious Diseases, St. John’s Research Institute, Bangalore, India; ^7^ Department of Infectious Diseases, Leiden University Medical Center, Leiden, Netherlands; ^8^ GlaxoSmithKline Vaccines, Wavre, Belgium; ^9^ Department of Infectious Diseases, Oslo University Hospital, Oslo, Norway

**Keywords:** transcript signature, protein signature, tuberculosis, adult, non-sputum

## Abstract

To achieve the ambitious targets for tuberculosis (TB) prevention, care, and control stated by the End TB Strategy, new health care strategies, diagnostic tools are warranted. Host-derived biosignatures are explored for their TB diagnostic potential in accordance with the WHO target product profiles (TPPs) for point-of-care (POC) testing. We aimed to identify sputum-independent TB diagnostic signatures in newly diagnosed adult pulmonary-TB (PTB) patients recruited in the context of a prospective household contact cohort study conducted in Andhra Pradesh, India. Whole-blood mRNA samples from 158 subjects (PTB, n = 109; age-matched household controls, n = 49) were examined by dual-color Reverse-Transcriptase Multiplex Ligation-dependent Probe-Amplification (dcRT-MLPA) for the expression of 198 pre-defined genes and a Mesoscale discovery assay for the concentration of 18 cytokines/chemokines in TB-antigen stimulated QuantiFERON supernatants. To identify signatures, we applied a two-step approach; in the first step, univariate filtering was used to identify and shortlist potentially predictive biomarkers; this step may be seen as removing redundant biomarkers. In the second step, a logistic regression approach was used such that group membership (PTB vs. household controls) became the binary response in a Lasso regression model. We identified an 11-gene signature that distinguished PTB from household controls with AUCs of ≥0.98 (95% CIs: 0.94–1.00), and a 4-protein signature (IFNγ, GMCSF, IL7 and IL15) that differentiated PTB from household controls with AUCs of ≥0.87 (95% CIs: 0.75–1.00), in our discovery cohort. Subsequently, we evaluated the performance of the 11-gene signature in two external validation data sets viz, an independent cohort at the Glenfield Hospital, University Hospitals of Leicester NHS Trust, Leicester, UK (GSE107994 data set), and the Catalysis treatment response cohort (GSE89403 data set) from South Africa. The 11-gene signature validated and distinguished PTB from healthy and asymptomatic *M. tuberculosis* infected household controls in the GSE107994 data set, with an AUC of 0.95 (95% CI: 0.91–0.98) and 0.94 (95% CI: 0.89–0.98). More interestingly in the GSE89403 data set, the 11-gene signature distinguished PTB from household controls and patients with other lung diseases with an AUC of 0.93 (95% CI: 0.87–0.99) and 0.73 (95% CI: 0.56–0.89). These criteria meet the WHO TTP benchmarks for a non–sputum-based triage test for TB diagnosis. We suggest that further validation is required before clinical implementation of the 11-gene signature we have identified markers will be possible.

## Introduction

Tuberculosis (TB) is one of the top 10 causes of death worldwide and the single infectious pathogen responsible for the most deaths–even after the emergence of the covid-19 pandemic. In 2018, a total of 1.5 million lives were lost to TB (1), and the goals of the End TB Strategy; to achieve a 90% reduction in TB incidence and a 95% reduction in TB mortality by 2035, are challenging (2). Much of the mortality attributed to TB occur in low-resource settings, so effective diagnostic tests applicable in these settings are essential to meet these goals. The WHO has defined the performance and operational characteristics of tests applicable for primary care or at the point-of-care (POC) in its high-priority target product profiles (TPPs) (3). To meet the TPPs, a rapid biomarker-based test would ideally be i) instrument-free or feasible with limited instrumentation and ii) based on easily accessible samples such as blood, urine, or breath (4).

In recent years, efforts have been made to identify which of the diagnostic needs should be of highest priority for biomarker-based assays balancing efficiency, affordability, and access in high-endemic limited resource settings (3, 5). The top priority is a rapid biomarker-based, non-sputum POC test i) to detect TB disease and guide immediate initiation of TB treatment, thus avoiding loss of cases from diagnostic delay (3, 5), and ii) for triage, ruling out TB disease with high sensitivity, allowing targeted referral to more expensive and accurate confirmatory tests (6). Ideally, such POC tests would perform satisfactorily with pulmonary and extrapulmonary disease in both children and adults regardless of HIV coinfection (7). In recent years, there have been exciting developments, including sputum-based and non-sputum-based TB diagnostics. However, the Lipoarabinomannan (LAM) test, which detects *M. tuberculosis (Mtb)* complex LAM in urine, is hitherto the only non-sputum test endorsed by WHO.

Over the past few years, the search for host biomarker(s) or biosignatures has gained increased attention in attempts to develop companion diagnostic platforms (8–20). Although expensive and resource-demanding, genome-wide analyses of transcriptomes offer unbiased identification of genes and immunologic pathways relevant for the understanding of TB pathogenesis, and risk of progression to disease (21–23). In the search for a unifying signature, a landmark study by Sweeney et al. (24) using data from publicly available human genome repositories, identified a 3-gene signature (3-gene TB score) derived from three discovery datasets of adults, that separated subjects with TB from healthy controls, *Mtb* infection, and other diseases in validation datasets of children and adults. However, the mean diagnostic accuracy obtained in the validation sets did not meet initial WHO criteria for a diagnostic POC test. Subsequently, Warsinke HC et al. (25) evaluated the performance of the 3-gene TB score in three different TB cohorts (25–27) and found that outcomes approached the WHO TTP benchmarks for a non-sputum-based triage test, with a high negative predictive value. Further, a very recent study evaluated 27 eligible identified signatures in a systematic meta-review, from which four signatures (Sweeney3, Kaforou25, Roe3, and BATF2) fulfilled the WHO minimum diagnostic accuracy parameters required for a TB triage test (28).

Genome-wide analysis of transcriptomes has been applied as a first step in identifying markers with potential for subsequent refinement as POC tests (12). To simplify the search for transcriptional signatures with diagnostic relevance in TB, we applied a user-friendly and inexpensive technique; the dual-color-Reverse-Transcriptase-Multiplex-Ligation-dependent-Probe-Amplification (dc-RT MLPA). In addition, a Mesoscale discovery assay was applied for protein analysis. The present study aimed to: i) Identify transcriptional and proteomic signatures with the ability to distinguish pulmonary TB (PTB) from household controls. ii) Validate the identified transcriptional signature in an independent cohort from the UK (17) comprising adult TB patients and healthy household contacts with/without *Mtb* infection as well as in the South African *Catalysis Treatment Response Cohort* (CTRC) (27) comprising adult TB patients, subjects with other lung diseases, and healthy controls. iii) Investigate the performance of the signature in adult TB patients identified in the present study in a recently-described pediatric population (11). iv) Compare the diagnostic abilities of the previously identified 10-gene signature (11) for pediatric PTB in the present adult study population.

## Materials and Methods

### Ethical Consideration

Ethical approval for this study was obtained from the Institutional Ethical Review Board (IERB) of St. John’s Medical College, Bangalore (IERB/1/527/08). The material transfer agreement between St. John’s Medical College, Bangalore, and the University of Bergen, Norway was obtained from the Department of Biotechnology, Government of India (No.BT/Med.II/Adv (SS)/Misc./02/2012). Ethical approval was also obtained (Ref no: 2018/1614 D) from the Regional Committee for Medical and Health Research Ethics, Western Norway.

### Study Population

A prospective cohort study of adult PTB index cases and their household contacts were conducted in Palamaner and Kuppam Taluks, Chittoor district, Andhra Pradesh, India (3.200°N, 72.7500°E, altitude 683 m) between September 2010 and April 2012. In total, 176 index cases were identified at the microscopy centres of the Revised National Tuberculosis Control Program (RNTCP) (Government of India). Of these, 164 were recruited following written informed consent, and 150 had confirmed TB (presence of *Mtb* in sputum smear and/or culture) with/without abnormal chest X-rays. All were treated with standard anti-TB treatment (ATT) and followed until the end of the 6-month ATT course. Household contacts of the 176 index cases were asked to participate and 525 household contacts recruited following written informed consent were followed for one year. For all children parents/guardians gave their written informed consent to participation. For participants >7 years, an additional written assent was obtained.

### Clinical Assessments and Sampling

*Baseline Assessments of PTB Index Cases and Household Contacts:* Medical History (including BCG vaccination status, history of TB exposure, prior TB/TB treatment and habitual risk factors), demographic, anthropometric, and clinical data were recorded. At baseline, a tuberculin skin test (TST) was performed by a trained nurse (2 TU/0.1 ml of tuberculin; Span Diagnostics, Surat, India) and read after 48–72 h; an induration ≥10 mm was defined as positive. Three independent radiologists interpreted the chest X-ray (anteroposterior view) at baseline. Agreement by at least two radiologists was required for a conclusion of findings suggestive of TB. Although not a pre-requisite for participation, HIV testing was performed in consenting subjects following pre-test counseling.

### External Validation Cohort

Gene expression data from the Singhania A et al; GSE107994 (an independent cohort of PTB and close contacts of household at the Glenfield Hospital, University Hospitals of Leicester NHS Trust, Leicester from UK) (17) and Thompson EG et al; GSE89403 (CTRC from South Africa) (27) data sets were used for external validation. The normalized log 2 data were back-transformed and multiplied by 100, to match the expression level with the dcRT-MLPA assay).

Gene expression data from our previous pediatric TB cohort (11) was used for validation. In addition, the 10-gene signature originally identified in the pediatric cohort (which consists of TB cases and asymptomatic TB-exposed household controls) was evaluated in the present adult PTB study cohort.

For the external validation, no proteomic data from TB-antigen (ag) stimulated QuantiFERON (QFT) supernatants were available for the proteomic signature evaluation.

### Sample Collection, RNA Extraction, and Selection of Transcriptional Biomarkers

Peripheral whole blood (approx. 2.5 ml) was drawn into PAXgene blood RNA tubes (PreAnalytiX, Hombrechtikon, Switzerland) and stored at -80°C until RNA extraction (PAXgene Blood RNA kit; PreAnalytiX, Hilden, Germany). Total RNA concentration and purity were measured using a Nanodrop spectrophotometer (Thermoscientific, Wilmington, DE, USA) and ranged between 0.4 –13.2µg (average 3.8 ± 1.65µg).

A total of 198 genes (including 4 housekeeping genes), distributed in 3 panels were assessed, based primarily on their posited or confirmed roles in TB immunology; the first 48-gene set (identified by the partners in the Bill and Melinda Gates Foundation Grand Challenge project #6 consortium) has been described in our previous studies (10, 13). The second 92-gene set included genes known to be involved in general inflammation and myeloid cell activation, and genes involved in the adaptive immune system, comprising Th1/Th2-responses, regulatory T-cell markers, and B-cell associated genes. The third 58-gene set included type 1-interferon-inducible genes (21) known to be up-regulated in adult TB and genes associated with prediction of TB risk in South African neonates (29). In total, thirty genes were present in more than one panel. For the 30 repeated genes that were present in more than one panel, geometric mean expression was used as done in our previous studies (11, 30). In total, there were 145 unique genes were analyzed and presented in the **Supplementary Table 1** (11, 30).

### Dual-Color-Reverse-Transcriptase-Multiplex-Ligation-Dependent-Probe-Amplification (dcRT-MLPA)

For each target sequence, a specific RT primer was designed, located immediately downstream of the left- and right-hand half-probe target sequence. A total of 125 ng RNA was used for reverse transcription, applying MMLV reverse transcriptase (Promega, Madison, WI, USA), followed by hybridization of left- and right-hand half-probes to the cDNA at 60° C overnight. The remaining steps were performed as described elsewhere (13, 31). All 158 samples were run in two (96-well) plates for each of the gene panels. The PCR fragments were analyzed on a 3730-capillary sequencer in Gene scan mode (Life Technologies, Carlsbad, CA, USA), using GeneMapper version 5.0 (Life Technologies, Carlsbad, California, USA). Primers and probes were obtained from the Department of Infectious Diseases, Leiden Medical University, the Netherlands. GAPDH was used for normalization.

### Multiplex Cytokine/Chemokine Assays

Biomarkers at the proteomic level were analyzed in peripheral whole blood stimulated with a mixture of *Mtb*-specific antigens (e.g., QFT supernatants): Early Secretory Antigenic Target-6 (ESAT-6), Culture Filtrate Protein-10 (CFP-10) and TB antigen 7.7. A pilot study was conducted on 12 randomly selected baseline samples from TB Cures (n = 4), Treatment Failures (n = 4) and household controls (n = 4) using the V-plex human pro-inflammatory, cytokine, and chemokine panels from Meso Scale Discovery (MSD, Rockville, Maryland, USA) according to the manufacturer’s instructions. Six of ten biomarkers from each panel [pro-inflammatory panel (IL1β, IL10, IL4, IL12p70, IFNγ, and TNFα), cytokine panel (GM-CSF, IL15, IL17A, IL5, IL7, and VEGF), and chemokine panel (Eotaxin3, IL8, IP10, MCP1, MDC, and MIP1β)] were analyzed. The analysis of biomarkers at the proteomic level has been described elsewhere (30).

### Data Analysis

Patient characteristics were summarized using mean and minimum/maximum or count and percentage, as appropriate. TB disease and household controls were compared using the Mann-Whitney test, Pearson’s chi-square test with Yates Continuity Correction, or Fisher’s exact test, as appropriate.

Both PTB cases (n = 48) and age-matched household controls irrespective of *Mtb* infection status (n = 49) were randomly divided into a training set (2/3), and a test set (1/3). Signatures were identified by means of a two-step approach previously used for biosignature identification (11). In short, the approach consisted of 1) univariate feature selection analysis using logistic regression, selecting markers by applying stringent p-value (p<0.01), and LASSO regression analysis based on the markers identified in step 1. The resulting LASSO model fits provided estimated coefficients (not reported in the present study, see Sivakumaran et al. (30) for an example). The model fits also enabled prediction of the probability of being a PTB for each participant. A predicted probability of >0.5 resulted in classification as a PTB case and <0.5 resulted in classification as a control. This model-based classification was compared to the actual “true” classification of participants and the number of correctly classified participants could be identified. Specifically, the predictive abilities of the signatures (to classify participants correctly) in both training and test set were summarized by means of receiver operator characteristic (ROC) curves, specifically sensitivity, specificity, and area under the curve (AUC). Analyses were carried out using R (R Core Team) (32) through the graphical user interface RStudio (www.rstudio.com).

## Results

### Baseline Clinical Characteristics of the PTB Index Cases

**Figure 1:**
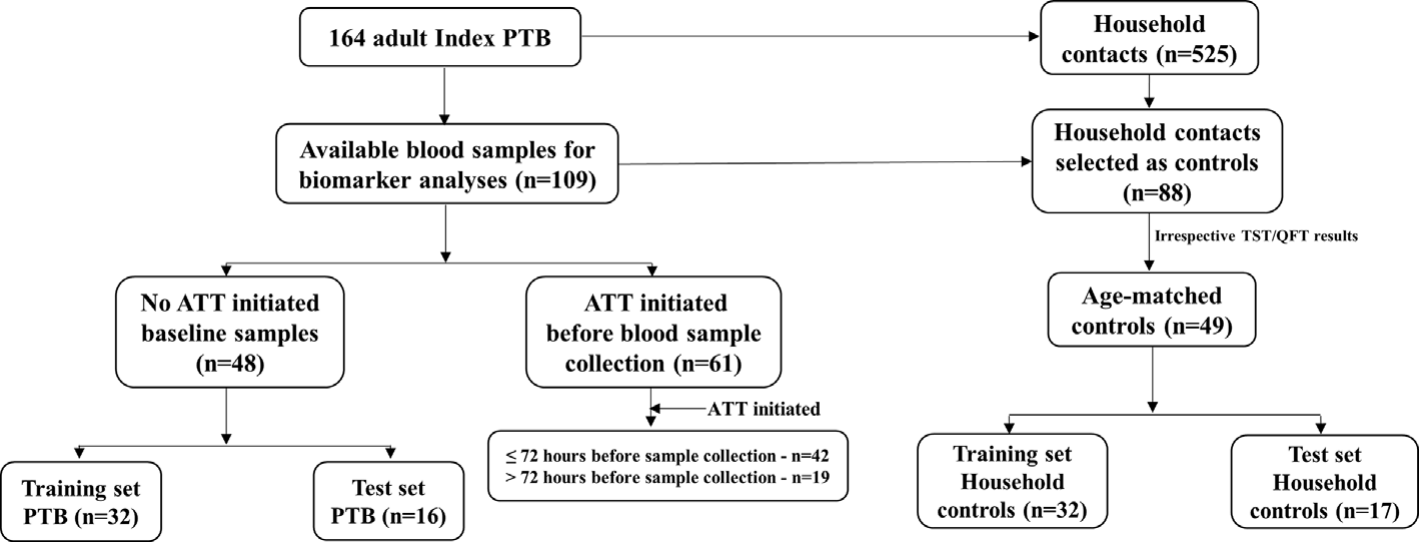
Study flow chart. PTB, pulmonary tuberculosis; ATT, anti TB therapy.

Blood samples at baseline were obtained from 109 of the 150 participants with confirmed TB, but only 48 were collected before ATT initiation and thus selected for further biomarker analysis. The remaining PTB (n = 61) cases were stratified based on timepoint for sample collection after ATT initiation (≤72 or >72 h) and analyzed separately (**Figure 1**). In the training set, the mean age was 43.9 years in PTB cases (range: 19–70) and 35.7 years in household controls (range: 18–80), and in the test set, 46.5 years in PTB cases (range: 19–69) and 38.2 years (range: 19.5–65) for household controls. In the training set, males constituted 90.6% (29/32) of PTB cases, and 31.3% (10/32) of household controls (p<0.001); in the test set, males constituted 75.0% (16/17) of PTB cases and 23.5% (4/17) of household controls (p<0.01; **Table 1**). Further baseline characteristics are shown in **Table 1**.

**Table 1:** Baseline characteristics of discovery data sets.

Clinical Characteristics	Training set		p-value	Test set		p-value
	TB disease (n = 32)	Household controls (n = 32)		TB disease (n = 16)	Household controls (n = 17)	
**Demographics**						
Age in years (mean)	43.9	35.7	**0.012**	46.5	38.2	0.1
Range	19–70	18–80		19–69	19.5–65	
Gender (Male)	29	10	**<0.001**	12	4	**0.003**
**Mycobacterial exposure**						
Known BCG vaccination	12	15	0.6	8	11	0.65
Unknown	4	5		2	1	
**Tuberculin skin test**						
Positive (≥10 mm)	26	17	**0.02**	11	9	0.5
Median (mm)	16.8	14.2		15.5	15.3	
**QuantiFERON Gold in tube**						
Positive (≥0,35 IU/ml)	24	19	0.14	12	8	0.09
Indeterminate	1	1		0	0	
Median (IU/ml)	3.05	2.78		5.07	8.2	
**Symptoms**						
Cough >2 weeks	29	0	**<0.001**	16	0	**<0.001**
Fever >1 week	25	1	**<0.001**	13	0	**<0.001**
Weight loss	22	0	**<0.001**	11	0	**<0.001**
**Findings**						
Abnormal Chest X-ray	31	0	**<0.001**	16	0	**<0.001**
BMI < 18.5 (under weight)	23	10	**0.001**	10	11	0.89

The significant p-values are in bold.

The mean age of the UK cohort was 40.3 (range: 20–75), 39.6 (range: 16–72), and 35.2 (range: 16–79) years for PTB, healthy *Mtb* infected household contacts, and contacts, respectively. Males constituted 67.9%, 57.1%, and 60% of each cohort. For PTB cases in the CTRC cohort the mean age was 33 years (range:: 17–66) and males constituted 65.0%.

### Identification of an 11-Gene Signature

The mean expression of unique 145 transcriptional biomarkers (in arbitrary units) are shown in **Supplementary Table 1**. We identified an 11-gene signature, comprising *CASP8*, *CD3E*, *CD8A*, *CD14*, *GBP5*, *GNLY*, *NLRP2*, *NOD2*, *TAGAP*, *TLR5*, and *TNF* (**Table 2A**) able to distinguish PTB cases from household controls with an AUC of 0.99 and 0.98 in the training and test sets, respectively (**Table 3A**).

**Table 2A:** Expression and regression coefficients for each biomarker of the identified 11-gene signature.

TB disease expression	Genes	Regression co-efficient
Increased	CD14	6.24E-05
	GBP5	4.92E-05
	NOD2	5.12E-04
	TLR5	4.22E-04
Decreased	CASP8	-1.41E-04
	CD3E	-2.65E-04
	CD8A	-1.83E-04
	GNLY	-2.16E-06
	NLRP2	-2.31E-04
	TAGAP	-2.59E-04
	TNF	-2.15E-03

**Table 3A:** Identification and performance of 11-gene signature.

Data sets	Sensitivity (95% CI)	Specificity (95% CI)	AUC (95% CI)	Accuracy in %
**PTB vs. Household controls**		** **	** **	** **
**Training set**	93.8 (77.8–98.9)	100.0 (86.7–100.0)	0.99 (0.99–1.00)	96.9
**Test set**	100 (75.9–100.0)	88.2 (62.3–97.9)	0.98 (0.94–1.00)	93.9
**PTB vs. Household controls**				
**Validation set 1**	77.4 (63.5–87.3)	92.0 (79.9–97.4)	0.95 (0.91–0.99)	84.5
**PTB vs. *Mtb* infected**				
**Validation set 2**	77.4 (63.5–87.3)	89.8 (77.0–96.2)	0.94 (0.89–0.98)	83.3
**PTB vs. Healthy controls**				
**Validation set 3**	52.6 (42.2–62.8)	95.2 (75.1–99.7)	0.93 (0.87–0.99)	60.3
**PTB vs. Other lung diseases**				
**Validation set 4**	52.6 (42.2–62.8)	82.4 (55.8–95.3)	0.73 (0.56–0.89)	57.1
**PTB vs. Household/asymptomatic controls**				
**Validation set 5**	36.2 (23.1–51.5)	94.4 (80.0–99.0)	0.69 (0.57–0.80)	61.4

Subsequently, we tested the performance of this 11-gene signature in PTB index cases ≤72 h and >72 h after ATT initiation as prior work suggested that in some cases gene expression can change significantly within first week of treatment (33). In this case, the results showed that ≤72 h after ATT-initiation, the TB cases had a similar, or marginally lower AUC (0.97, 95% CI, 0.94–1.00) compared to the >72 h ATT-initiated TB cases (AUC = 0.99; 95% CI, 0.99–1.00).

### Evaluation of the Identified 11-Gene Signature in Other Data Sets

#### Study 1: Singhania A et al.; GSE107994 Adult Data Set

**Figure 2:**
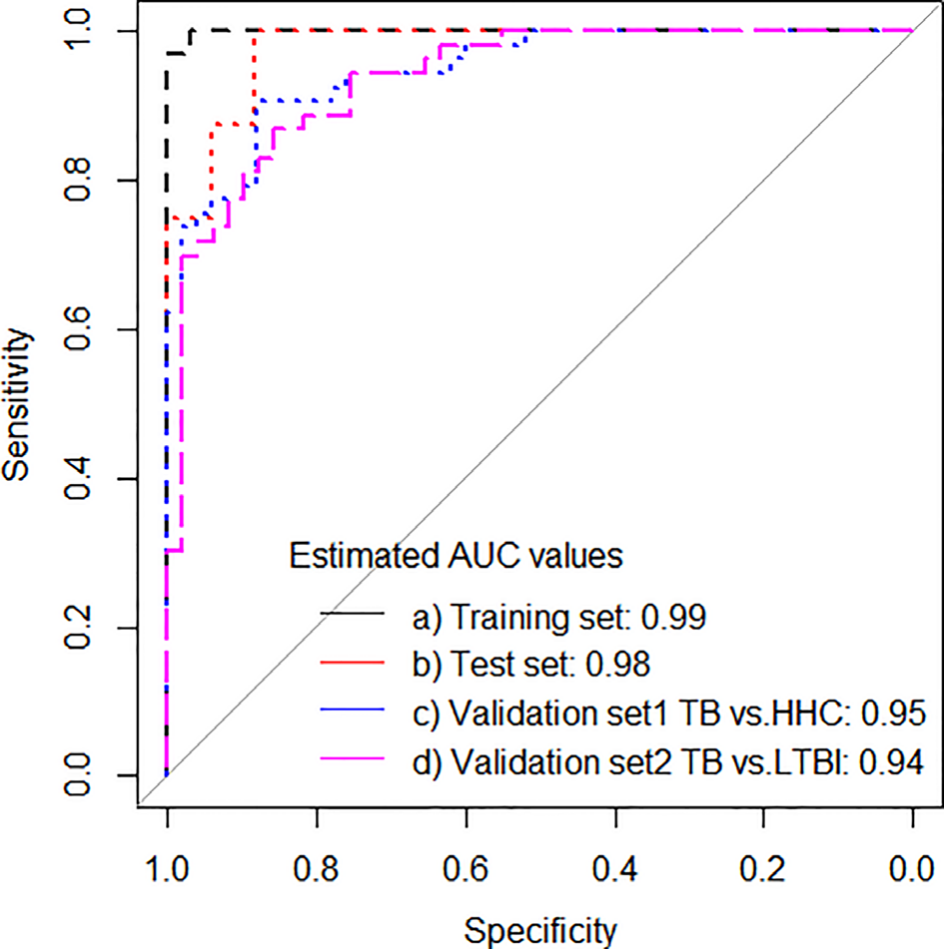
ROC curves for signature that distinguishes PTB from household controls in the training set, test set, whereas in validation set 1 (ATB vs. healthy recent contacts) and validation set 2 (ATB vs. LTBI).

The performance of the 11-gene signature was then evaluated in the GSE107994 data set (UK cohort as validation set 1 and 2), which provided an AUC of 0.95 (95% CI: 0.91–1.00) correctly classifying 41 of 53 PTB (sensitivity 77.4%, 95% CI, 63.5–87.3), and 46 of 50 healthy *Mtb*-uninfected household contacts (specificity 92.0%, 95% CI, 79.9–97.4). Similarly, the 11-gene signature differentiated PTB from *Mtb*-infected household contacts with an AUC of 0.94 (95% CI: 0.89–0.98), with a specificity of 89.8% (95% CI, 77.0–96.2; **Table 3A** and **Figure 2**).

#### Study 2: Thompson EG et al.; GSE89403 Adult Data Set

**Figure 3:**
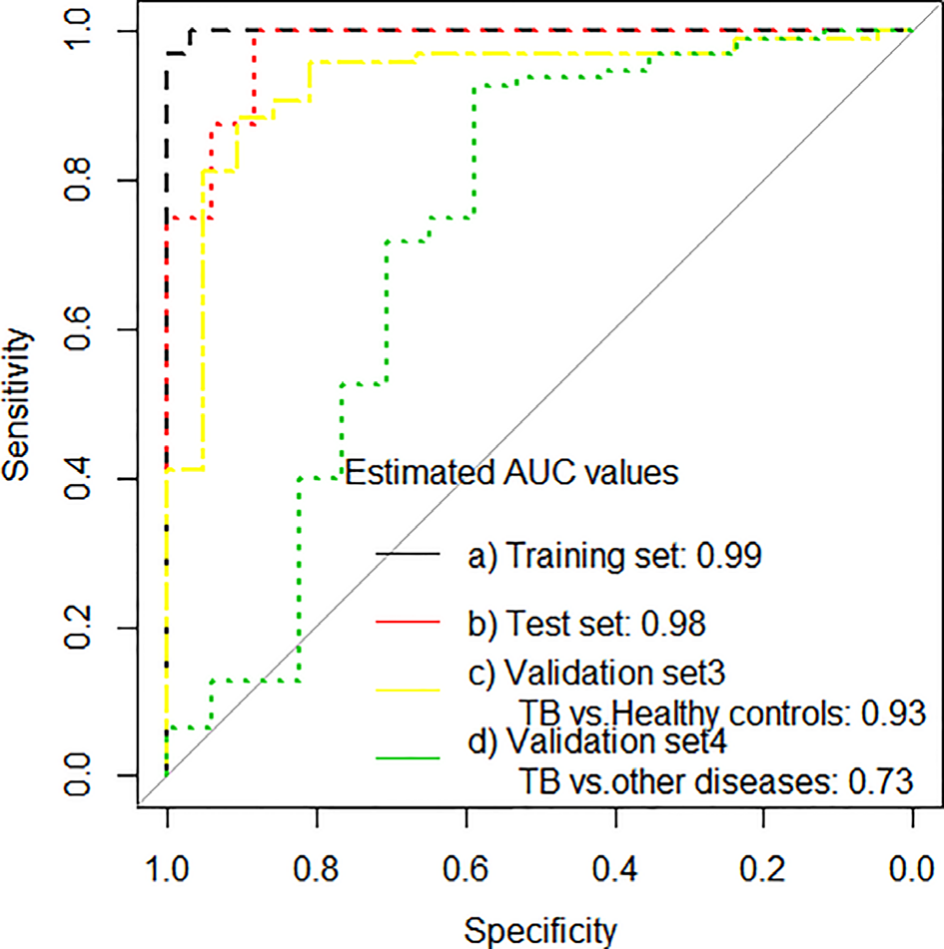
ROC curves for signature that distinguishes PTB from household controls in the training set, test set and in validation set 3 (PTB from healthy controls) and validation set 4 (PTB from other lung diseases).

The performance of the 11-gene signature was also evaluated in the GSE89403 data set (South African CTRC as validation set 3 and 4), where it gave an AUC of 0.93 (95% CI: 0.87–0.99) correctly classifying 50 of 95 PTB cases (sensitivity 52.6%, 95% CI, 42.2–62.8), and 20 of 21 healthy controls (specificity 95.2%, 95% CI, 75.1–99.7). Interestingly, given the real-life diagnostic challenges faced in differentiating TB patients from other symptomatic patients, the 11-gene signature differentiated PTB from other lung diseases with an AUC of 0.73 (95% CI: 0.56–0.89), with a specificity of 82.4% (95% CI, 55.8–95.3; **Table 3A** and **Figure 3**).

#### Study 3: JE Gjøen et al.; Pediatric Data Set

**Figure 4:**
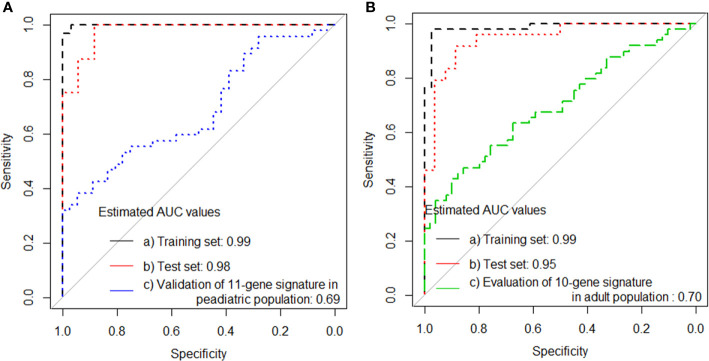
ROC curves for signature that distinguishes PTB from household controls **(A)** the training set, test set, validation of adult 11-gene signature in the pediatric population and **(B)** the training set, test set, validation of pediatric10-gene signature in the adult population.

Finally, the performance of the 11-gene signature was evaluated in a pediatric data set collected previously by our group (validation set 5), presented an AUC of 0.69 (95% 0.57–0.80), which correctly classified 17 of 47 PTB (sensitivity 36.2%, 95% CI, 23.1–51.5), and 34 of 36 household controls (specificity 94.4%, 95% CI, 80.0–99.0; **Table 3A** and **Figure 4A**).

### Evaluation of Our Pediatric 10-Gene Signature in the Adult TB Population in the Present Study

As the 11-gene signature identified in adults performed poorly in children, we asked if our previously identified diagnostic 10-gene pediatric signature would perform better in our adult PTB cases, but the AUC of 0.70 (95% CI, 0.60–0.80) obtained was similar to validation set 5 (**Figure 4B**).

### Identification of Proteomic Signature

**Figure 5:**
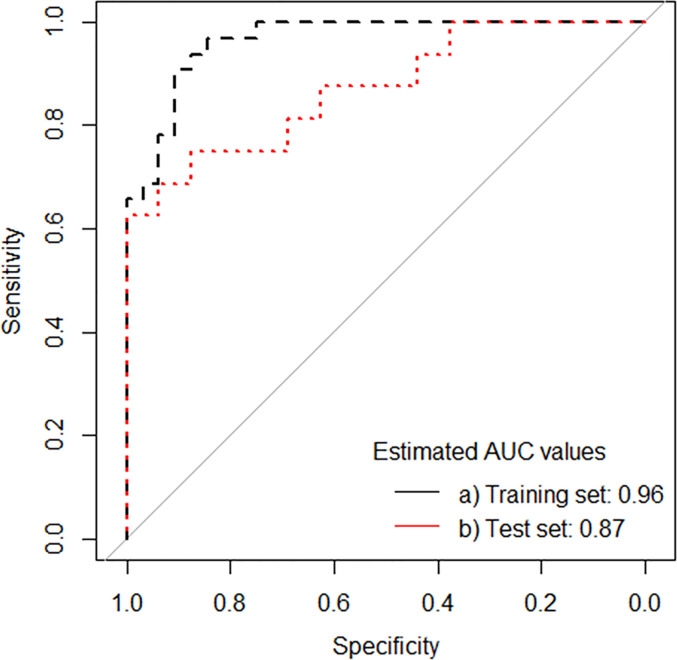
ROC curves for protein signature that distinguishes PTB from household controls in the training set and test set.

The median concentration (pg/ml) of the 18 protein biomarkers measured are shown in **Supplementary Table 2**. We applied Lasso regression analysis directly on data from the 18 protein biomarkers tested, and identified a 4-protein signature, comprising IFNγ, GMCSF, IL7, and IL15 (**Table 2B**) that differentiated PTB from healthy household controls with an AUC of 0.96 (95% CI, 0.92–1.00) in the training set, correctly classifying 28 of 32 PTB cases (sensitivity 87.5%, 95% CI, 70.1–95.9), and 29 of 32 household controls (specificity 90.6%, 95% CI, 73.8–97.5). In the test set, the identified signature generated an AUC of 0.87 (95% CI, 0.75–0.99), correctly classifying 11 of 16 PTB cases (sensitivity 68.8%, 95% CI, 41.5–87.9), and 16 of 17 household controls (specificity 94.1%, 95% CI, 69.2–99.7; **Table 3B** and **Figure 5**).

**Table 2B:** Expression and regression coefficients for each biomarker of the identified 4-protein signature.

TB disease expression	Proteins	Regression co-efficient
Increased	IL7	2.07E+00
	IL15	7.87E-02
Decreased	IFNγ	-1.10E-06
	GMCSF	-7.65E-03

**Table 3B:** Identification of 4–protein signature.

PTB vs. Household controls				
**Training set**	87.5 (70.1–95.9)	90.6 (73.8–97.5)	0.96 (0.92–1.00)	89.1
**Test set**	68.8 (41.5–87.9)	94.1 (69.2–99.7)	0.87 (0.75–0.99)	81.8

Similarly, we tested the performance of the 4-protein signature in the ATT-initiated participants vs. household controls. PTB cases initiated on ATT ≤72 prior to sampling had a slightly higher AUC value (0.89, 95% CI, 0.81–0.97) compared to PTB cases initiated on ATT >72 h prior to sampling (AUC = 0.88; 95% CI, 0.75–0.99).

## Discussion

An ideal diagnostic biomarker or multiple marker biosignature for TB could be either pathogen- or host-derived and should be specific to the underlying disease process (4, 34). Several transcriptional signatures based on testing in different ethnic populations have been proposed for this purpose by numerous research groups (14, 17, 21, 24, 35, 36). However, limited overlap in genes differentially expressed between PTB and household controls have been found when comparing these signatures. A recent meta-analysis identified eight signatures with an equivalent performance that showed moderate to high correlation for diagnosing incipient TB. Overlapping constituent genes only partially accounted for correlation between signatures, suggesting that they reflect different dimensions of the typical host response to infection with *Mtb*, and strongly supported the identification of IFN and TNF signaling pathways as statistically enriched upstream regulators of the genes across the eight signatures (37). Several attempts have been made to reduce the large number of genes identified by these studies as potentially relevant into smaller candidate signatures that could form the basis of a potential clinical diagnostic. However, there is still no agreement as to which genes to include in an optimal diagnostic signature.

In this study, we report that our 11-gene whole blood transcriptomic signature gave promising diagnostic performance across diverse populations (India, UK, South Africa) from both low-endemic and high-endemic countries, based on a capacity to distinguish PTB from household controls with an AUC ≥ 0.93. However, the 11-gene signature was less successful in efficiently discriminating TB disease from other lung diseases. The evaluation of this 11-gene signature in the UK-derived cohort indicated reasonable diagnostic accuracy (> 80.0, **Table 3**) for the identification of PTB. However, in the CTRC cohort, the performance of the 11-gene signature was lower. Aiming for a POC triage test to ascertain targeted referral of symptomatic subjects in the field, this shortcoming in accuracy can to some extent be overcome by clinical algorithms that include reassessment and referral of subjects with lack of improvement from assumed intercurrent infections (with or without antibiotics dependent on clinical presentation). The reasons for discrepancy between the two cohorts are likely multifactorial reflecting differences in ethnicity, sample size, mean age (in years) and lack of other lung disease controls in our cohort. The transcriptional signature identified in the present study meets WHO TTP minimal requirement for a screening test, but further evaluation will be required before clinical implementation is possible.

Warsinske HC, et al. (25) have analyzed the performance of the 3-gene TB score (*GBP5*, *DUSP3*, and *KLF2*) in three different TB cohorts. *i) South African adolescent cohort of TB progressors (age in years*, *12*–*18)*: those who progressed from latent *Mtb* infection to PTB compared with non-progressors (26), *ii) Brazil Active Screening Study Cohort (age in years, 18*–*80)*: all positive sputum culture for *Mtb* compared with controls that were sputum culture-*negative* (25), *iii) South African CTR Cohort (age in years, 17*–*66): comprises culture-positive* patients with PTB, healthy controls, and patients with other lung diseases (pneumonia or asthma). PTB patients all received standard treatment following diagnosis (27). Across all three cohorts, at a TB disease prevalence of 4%, the 3-gene TB score identified TB patients with a 90% sensitivity, a specificity of 70%, and a negative predictive value of 99.3% (25). Notably, the *GBP5* gene was also up-regulated and is included in our pediatric 10-gene and adult 11-gene signatures. Besides, *GBP5* was also reported previously by Esterhuyse MM (38) and Zak DE (26) et al. These findings suggest that *GBP5* could be a potential component in a unified biomarker signature for TB.

Previous studies have identified different transcript signatures for distinguishing TB from latent TB and other diseases in Malawian and South African pediatric (35) and adult (14) cohorts, which could highlight the differences in pathogenesis of adult versus pediatric TB. This is consistent with the findings from the present study where the adult biosignature’s poor performance in the pediatric cohort and vice versa suggests that it may be challenging to find a universally-applicable POC triage test for TB. This is despite the fact that the differentially expressed genes (whether down or up-regulated) showed the same trend in both pediatric and adult populations. Despite decades of research, significant investment, and numerous reports on new biomarker candidates, few biomarkers have been independently validated for both clinical trials and routine clinical use, and translated into new diagnostic tests (39, 40). This problem is not unique to TB; it is true for biomarker research in general that very few of the identified biomarkers have advanced to approved diagnostic tests in clinical use.

Interestingly, 3 genes do overlap (*GBP5*, *NOD2*, and *CD3E*) between the pediatric 10-gene signature and the adult 11-gene signature. Of these, two genes were up-regulated (*GBP5* and *NOD2*) and one gene down-regulated (*CD3E*) in PTB disease compared to household controls. Notably, both signatures were identified in an Indian population recruited from the same area when applying the same dcRT-MLPA method. This method is sensitive, and has high-throughput, but gives limited transcriptional data compared to RNA sequencing. This may explain some of the lack of overlap with transcript signatures identified in other studies, as not all genes of interest reported in other studies were included in our pre-defined gene panels.

In recent years, there have been more studies attempted to identify protein signature for TB disease in adults (9, 18, 41, 42) and children (43). A recent study hypothesized that a blood protein-based host response signature for active PTB could discriminate it from other TB-like disease (OTD) in adult patients with persistent cough and provide the foundation for a community-based triage test for PTB. The study identified a host blood protein signature consisting of IL-6, IL-8, IL-18, and VEGF, that discriminated active PTB from OTD with an AUC of 0.80, corresponding to a sensitivity of 80% and a specificity of 65% (41). The present study also identified a 4-protein signature (IFNγ, GMCSF, IL15, and IL7) in TB-ag stimulated QFT supernatants that distinguishes PTB patients from their household controls with AUCs ≥ 0.87, providing proof of concept for a protein-based approach.

The present study has some limitations: i) No formal sample size calculation was carried out since the maximum sample size was defined by the availability of samples for biomarker analysis, a factor exacerbated by the need to divide the samples into training and test sets. To some extent, however, this limitation was offset by the use of multiple validation cohorts, as described; ii) Lack of validation in extra-pulmonary TB cases—a population in which non-sputum based diagnostics are strongly needed; iii) Inability to cross-validate the identified proteomic signature due to the lack of comparable samples from other cohorts. Although host-response-based diagnostics are believed to be less dependent on bacterial load, an obvious advantage for TB diagnosis, it is unclear if these tools can be further optimized to meet the WHO target for a universally applicable POC test. With the increasing number of blood-based signatures for TB diagnosis being proposed, it is crucial to pool data across cohorts’ diverse in geographic, genetic, demographic and endemic characteristics in order to diminish time and costs for POC test evaluation with regard to the WHO TPP, and subsequent validation prior to translation to clinical practice.

## Data Availability Statement

The raw data supporting the conclusions of this article will be made available by the authors, without undue reservation.

## Ethics Statement

Ethical approval for this study was obtained from the Institutional Ethical Review Board (IERB) of St. John’s Medical College, Bangalore (IERB/1/527/08). The material transfer agreement between St. John’s Medical College, Bangalore, and the University of Bergen, Norway was obtained from the Department of Biotechnology, Government of India (no. BT/Med.II/Adv (SS)/Misc./02/2012). Ethical approval was also obtained (ref no: 2018/1614 D) from the Regional Committee for Medical and Health Research Ethics, Western Norway. Written informed consent to participate in this study was provided by the participants’ legal guardian/next of kin.

## Author Contributions

DS, JG, SJ, MV, TMD, CR, and HMSG conceptualized and designed the biomarker study. SS and MV coordinated patient recruitment and follow-up. DS wrote the manuscript with contribution from MV, CR, JG, SJ, TMD, and HMSG. DS performed all laboratory experiments. DS performed the data analysis and generated Tables and Figures. CR supervised the statistical analysis, wrote the section on statistical analysis, and reviewed the manuscript. TO contributed to the study design and analysis and reviewed the manuscript. HMSG had primary responsibility for the final content of the manuscript. All authors contributed to the article and approved the submitted version.

## Funding 

Research Council of Norway Global Health and Vaccination Research (GLOBVAC) projects: RCN 179342, 192534, and 248042, the University of Bergen (Norway); EDCTP2 program supported by the European Union; the St. John’s Research Institute, Bangalore. We also acknowledge EC FP7 ADITEC (grant agreement no. 280873); EC HORIZON2020 TBVAC2020 (grant agreement no. 643381) [the text represents the authors’ views and does not necessarily represent a position of the Commission who will not be liable for the use made of such information].

## Conflict of Interest

TMD is an employee of and holds shares in the GSK group of companies but participated in the current work as an independent investigator.

The remaining authors declare that the research was conducted in the absence of any commercial or financial relationships that could be construed as a potential conflict of interest.
