# Supplementary material for: Host Blood RNA Transcript and Protein Signatures for Sputum-Independent Diagnostics of Tuberculosis in Adults
Source: Front Immunol. 2021 Feb 4;11:626049. doi: 10.3389/fimmu.2020.626049 (PMC7891042; doi:10.3389/fimmu.2020.626049)
Supplement: Supplementary file 1 [file DataSheet_1.pdf]

Supplementary Table 1: dcRT-MLPA assay gene panels

Gene	Gene Name	TB disease Mean expression (interquartile range)	Household controls Mean expression (interquartile range)
	**Panel A[1,2]**		
**ABR**	Active BCR-Related gene	4027 (3317-4567)	4655 (3797-5391)
**BCL2**	B-Cell CLL/Lymphoma 2	871 (482-1127)	1491 (1070-1762)
**BLR1**	Burkitt Lymphoma Receptor 1	954 (443-1212)	2298 (1732-2804)
**CASP8**	Cysteine-Aspartic Acid Protease 8	5486 (4310-6379)	7507 (6257-8629)
**CCL13**	C-C Motif Chemokine Ligand 13	200 (200-200)	200 (200-200)
**CCL19**	C-C Motif Chemokine Ligand 19	212 (200-220)	216 (200-240)
**CD4**	CD4 Molecule	3964 (2736-5121)	5154 (4287-5891)
**CD14**	CD14 Molecule	18439 (15754-20361)	14092 (11305-16390)
**CD19**	CD19 Molecule	402 (200-471)	833 (679-1007)
**CD163**	CD163 Molecule	746 (438-741)	543 (392-641)
**CTLA4**	Cytotoxic T-lymphocyte Antigen 4	1016 (755-1207)	1418 (1161-1621)
**FASLG**	Fas Ligand	200 (200-200)	200 (200-200)
**FOXP3**	Forkhead Box P3	351 (200-362)	341 (200-353)
**FPR1**	Formyl Peptide Receptor 1	26853 (19891-30962)	20140 (15655-23737)
**IL4**	Interleukin 4	200 (200-200)	200 (200-200)
**IL4d2**	Interleukin 4 Delta 2	200 (200-200)	200 (200-200)
**IL7R**	Interleukin 7 Receptor	6538 (3939-7847)	15466 (11827-17988)
**LTF**	Lacto-transferrin	669 (200753)	244 (200-275)
**MARCO**	Macrophage Receptor	216 (200-200)	200 (200-200)
**MMP9**	Matrix Metallo-Peptidase 9	1291 (712-1542)	612 (276-826)
**NCAM1**	Neural Cell Adhesion Molecule 1	382 (200-320)	348 (240-446)
**RAB13**	Ras Related Protein-13	352 (200-471)	1032 (673-1228)
**RAB24**	Ras Related Protein-24	4966 (3794-6159)	4865 (3817-5895)
**RAB33A**	Ras Related Protein-33A	230 (200-245)	275 (200-312)
**SEC14L1**	SEC14 Cytosolic Factor Family	14687 (1070818431)	13980 (10794-16694)
**SPP1**	Secreted Phosphoprotein 1	200 (200-200)	200 (200-200)
**TGFBR2**	Transforming Growth Factor Beta Receptor 2	4035 (3052-4692)	4742 (3806-5421)
**TIMP2**	Tissue Inhibitor of Metallo-proteinases 2	23346 (19905-25990)	21085 (17072-24635)
**TNFRSF1A**	TNF Receptor Superfamily Member 1A	17028 (14537-19053)	16497 (14299-18763)
**TNFRSF1B**	TNF Receptor Superfamily Member 1B	4927 (3880-5349)	4366 (3464-5077)
	**Panel B**		
**AIRE**	Autoimmune regulator	258 (200-276)	321 (200-337)
**AREG**	Amphiregulin	4180 (2978-5378)	4882 (3184-6355)
**BPI**	Bactericidal/Permeability-Increasing Protein	5481 (4051-7131)	7289 (5115-8857)
**CAMTA1**	Calmodulin Binding Transcription Activator 1	200 (200-200)	200 (200-200)
**CCL2**	C-C Motif Chemokine Ligand 2	200 (200-200)	233 (200-241)
**CCL22**	C-C Motif Chemokine Ligand 22	866 (503-1252)	1160 (720-1706)

**CCL4**	C-C Motif Chemokine Ligand 4	591 (427-740)	871 (710-1035)
**CCL5**	C-C Motif Chemokine Ligand 5	23559 (17682-27539)	35678 (28744-40328)
**CCR7**	C-C Motif Chemokine Receptor 7	791 (252-1040)	1936 (1216-2578)
**CD209**	CD209 Molecule	3707 (2818-4293)	5130 (4123-5818)
**CD3E**	CD3e Molecule	3408 (2413-4329)	7546 (6444-8887)
**CD8A**	CD8a Molecule	1473 (1004-1963)	3279 (2152-4033)
**CLEC7A**	C-Type Lectin Domain Family 7 Member A	11363 (7872-13153)	10515 (7418-12390)
**CXCL13**	C-X-C Motif Chemokine Ligand 13	1933 (1401-2449)	2695 (2298-3186)
**FCGR1A**	Fc Fragment Of IgG Receptor Ia	3251 (1919-4179)	832 (200-1104)
**FLCN1**	Folliculin	217 (200-228)	220 (200-232)
**GATA3**	GATA Binding Protein 3	200 (200-200)	230 (200-243)
**GNLY**	Granulysin	23321 (14752-28754)	47066 (33390-63663)
**GZMA**	Granzyme A	1124 (598-1457)	2290 (997-3075)
**GZMB**	Granzyme B	4081 (2214-5181)	6534 (4875-7568)
**IFNG**	Interferon Gamma	200 (200-200)	200 (200-200)
**IL2**	Interleukin2	200 (200-200)	200 (200-200)
**IL5**	Interleukin 5	684 (226-986)	1013 (553-1396)
**IL9**	Interleukin 9	686 (200-752)	716 (200-752)
**IL10**	Interleukin 10	222 (200-240)	206 (200-202)
**IL13**	Interleukin 13	337 (200-359)	477 (200-510)
**IL15**	Interleukin 15	200 (200-200)	200 (200-200)
**IL12A**	Interleukin 12A	526 ((331-691)	634 (465-800)
**IL17A**	Interleukin 17A	200 (200-200)	200 (200-200)
**IL23A**	Interleukin 23A	495 (200-633)	457 (200-647)
**IL1B**	Interleukin 1B	3822 (2651-4652)	2630 (2049-3090)
**IL12B**	Interleukin 12B	407 (295-487)	569 (413-672)
**LAG3**	Lymphocyte Activating 3	200 (200-200)	200 (200-200)
**MRC1**	Mannose Receptor C-Type 1	200 (200-200)	200 (200-200)
**MRC2**	Mannose Receptor C-Type 2	200 (200-200)	200 (200-200)
**NEDD4L**	Neural Precursor Cell Expressed, Developmentally Down-Regulated 4-Like, E3 Ubiquitin Protein Ligase	372 (200-506)	349 (200-425)
**NLRC4**	NLR Family CARD Domain Containing 4	1449 (1172-1635)	1272 (1110-1427)
**NLRP1**	NLR Family Pyrin Domain Containing 1	7122 (4772-8687)	10683 (8126-12580)
**NLRP2**	NLR Family Pyrin Domain Containing 2	1551 (944-2002)	3108 (2479-3669)
**NLRP3**	NLR Family Pyrin Domain Containing 3	493 (385-604)	529 (441-631)
**NLRP4**	NLR Family Pyrin Domain Containing 4	252 (200-293)	301 (200-366)
**NLRP6**	NLR Family Pyrin Domain Containing 6	221 (200-234)	206 (200-213)
**NLRP7**	NLR Family Pyrin Domain Containing 7	200 (200-200)	200 (200-200)
**NLRP10**	NLR Family Pyrin Domain Containing 10	200 (200-200)	200 (200-200)
**NLRP11**	NLR Family Pyrin Domain Containing 11	200 (200-200)	200 (200-200)
**NLRP12**	NLR Family Pyrin Domain Containing 12	656 (549-745)	778 (602-961)
**NLRP13**	NLR Family Pyrin Domain Containing 13	457 (200-521)	592 (200-633)
**NOD1**	Nucleotide Binding Oligomerization Domain Containing 1	1257 (958-1535)	1937 (1588-2264)
**NOD2**	Nucleotide Binding Oligomerization Domain Containing 2	996 (765-1120)	707 (582-868)

**PRF1**	Perforin 1	7338 (3694-9915)	11941 (9161-14803)
**PTPRCv1**	Protein tyrosine phosphatase receptor type Cv1	2991 (1852-3942)	5324 (4173-6503)
**PTPRCv2**	protein tyrosine phosphatase receptor type Cv2	11283 (8495-13620)	11061 (8680-12964)
**RORC**	RAR Related Orphan Receptor C	200 (200-200)	213 (200-220)
**TAGAP**	T-Cell Activation RhoGTPase Activating Protein	10934 (8758-12826)	15656 (14122-16490)
**TBC1D7**	TBC1 Domain Family Member 7	201 (200-204)	201 (200-201)
**TBX21**	T-Box 21	200 (200-200)	200 (200-200)
**TGFB1**	Transforming Growth Factor Beta 1	41732 (36603-45561)	46230 (41093-50473)
**TLR1**	Toll Like Receptor 1	5168 (3644-6304)	4673 (3382-5321)
**TLR2**	Toll Like Receptor 2	2360 (1895-2754)	1609 (1184-1883)
**TLR3**	Toll Like Receptor 3	200 (200-200)	200 (200-200)
**TLR4**	Toll Like Receptor 4	2763 (2169-3073)	2380 (1726-2802)
**TLR5**	Toll Like Receptor 5	760 (428-1002)	315 (200-416)
**TLR6**	Toll Like Receptor 6	2438 (1557-3349)	1910 (1243-2261)
**TLR7**	Toll Like Receptor 7	1272 (1077-1482)	1354 (1101-1500)
**TLR8**	Toll Like Receptor 8	2545 (602-3843)	2088 (904-2681)
**TLR9**	Toll Like Receptor 9	1530 (200-2231)	2304 (200-2492)
**TLR10**	Toll Like Receptor 10	200 (200-200)	200 (200-200)
**TNF**	Tumor Necrosis Factor	1219 (1036-1388)	1658 (1474-1816)
**TNFRSF18**	TNF Receptor Superfamily Member 18	200 (200-200)	200 (200-200)
**TWIST1**	Twist Family BHLH Transcription Factor 1	200 (200-200)	200 (200-200)
**ZNF331**	Zinc Finger Protein 331	218 (200-230)	253 (200-296)
**ZNF532**	Zinc Finger Protein 532	200 (200-200)	200 (200-200)
	**Panel C[3,4]**		
**ASAP1**	ArfGAP With SH3 Domain, Ankyrin Repeat And PH Domain 1	6444 (5364-7566)	6482 (5542-7202)
**B2M**	Beta-2-Microglobulin	60592 (39214-79293)	67009 (39972-83118)
**BMP6**	Bone Morphogenetic Protein 6	6796 (6136-7609)	9839 (8024-10589)
**CCL11**	C-X-C Motif Chemokine Ligand 11	11975 (9451-14024)	17420 (13431-19963)
**CCL3**	C-C Motif Chemokine Ligand 3	1294 (819-1621)	1990 (1547-2341)
**CD274**	CD274 Molecule	2374 (501-3536)	276 (200-318)
**CX3CL1**	C-X3-C Motif Chemokine Ligand 1	202 (200-204)	212 (200-224)
**CXCL9**	C-X-C Motif Chemokine Ligand 9	200 (200-200)	200 (200-200)
**CXCL10**	C-X-C Motif Chemokine Ligand 10	781 (563-965)	1082 (799-1202)
**DSE**	Dermatan Sulfate Epimerase	1075 (904-1285)	1006 (882-1109)
**EGF**	Epidermal Growth Factor	204 (200-208)	218 (200-231)
**GBP1**	Guanylate Binding Protein 1	2698 (1539-3294)	1094 (200-1521)
**GBP2**	Guanylate Binding Protein 2	43458 (31241-49195)	23147 (16168-29187)
**GBP5**	Guanylate Binding Protein 5	52977 (35400-67552)	21766 (14328-23331)
**GUSB**	Glucuronidase Beta	1804 (1380-2131)	2717 (2258-3223)
**HCK**	HCK Proto-Oncogene, Src Family Tyrosine Kinase	20720 (18105-23194)	20863 (18979-22401)
**HPRT**	Hypoxanthine Phosphoribosyltransferase 1	200 (200-200)	200 (200-200)
**IFI6**	Interferon Alpha Inducible Protein 6	16033 (9553-20760)	18573 (10325-20602)
**IFI16**	Interferon Gamma Inducible Protein 16	36791 (32438-39933)	42896 (36882-50051)

**IFI35**	Interferon Induced Protein 35	12533 (10659-14186)	15807 (13163-17412)
**IFI44**	Interferon Induced Protein 44	4080 (1661-5395)	4847 (1414-5020)
**IFI44L**	Interferon Induced Protein 44 Like	4263 (1704-5506)	4392 (719-4356)
**IFIH1**	Interferon Induced With Helicase C Domain 1	2734 (1961-3238)	2516 (1619-2851)
**IFIT2**	Interferon Induced Protein With Tetratricopeptide Repeats 2	15786 (9157-21319)	16713 (7482-17331)
**IFIT3**	Interferon Induced Protein With Tetratricopeptide Repeats 3	10951 (6175-14092)	11943 (5198-11974)
**IFIT5**	Interferon Induced Protein With Tetratricopeptide Repeats 5	5739 (3470-7824)	6178 (3795-6747)
**IFITM3**	Interferon Induced Transmembrane Protein 3	11091 (5968-15583)	5524 (1831-6460)
**IL6**	Interleukin 6	2690 (2163-3074)	4052 (3364-4782)
**INDO**	Indoleamine 2,3-Dioxygenase 1	215 (200-236	294 (200-347)
**IRF7**	Interferon Regulatory Factor 7	200 (200-200)	200 (200-200)
**KIF1B**	Kinesin Family Member 1B	3591 (3171-3998)	3563 (3182-3819)
**LYN**	LYN Proto-Oncogene, Src Family Tyrosine Kinase	17575 (14855-19029)	15760 (13246-18587)
**OAS1**	2'-5'-Oligoadenylate Synthetase 1	3951 (1832-4180)	4425 (1630-4177)
**OAS2**	2'-5'-Oligoadenylate Synthetase 2	852 (200-1011)	1023 (200-1147)
**OAS3**	2'-5'-Oligoadenylate Synthetase 3	2532 (986-3045)	2848 (873-2337)
**SLAMF7**	SLAM Family Member 7	1969 (1534-2336)	2323 (1981-2696)
**SOCS1**	Suppressor Of Cytokine Signaling 1	640 (200-885)	520 (200-712)
**STAT1**	Signal Transducer And Activator Of Transcription 1	10988 (8418-13105)	8690 (5886-10529)
**STAT2**	Signal Transducer And Activator Of Transcription 2	2087 (1554-2486)	1909 (1269-2280)
**TAP1**	Transporter 1, ATP Binding Cassette Subfamily B Member	12569 (9645-15306)	8827 (6331-10172)
**TAP2**	Transporter 2, ATP Binding Cassette Subfamily B Member	3575 (2666-4457)	3246 (2444-3930)
**TNIP1**	TNFAIP3 Interacting Protein 1	2579 (2187-2924)	2512 (2123-2763)
**VEGF**	Vascular Endothelial Growth Factor A	294 (200-378)	335 (200-452)

The genes and the gene names and the distiribusion to the 3 gene panels selected for the study.
The repeated genes (genes present in more then one panel) are presented in the panel where the gene gave the highest mean expression in all study subjects.

References:

1. Joosten, S. A. *et al.* Identification of biomarkers for tuberculosis disease using a novel dual-color RT-MLPA assay. *Genes Immun* 13, 71-82, doi:10.1038/gene.2011.64 (2012)

2. Dhanasekaran, S. *et al.* Identification of biomarkers for Mycobacterium tuberculosis infection and disease in BCG-vaccinated young children in Southern India. *Genes Immun* 14, 356-364, doi:10.1038/gene.2013.26 (2013)

3. Berry, M. P. et al. An interferon-inducible neutrophil-driven blood transcriptional signature in human tuberculosis. Nature 466, 973-977, doi:10.1038/nature09247 (2010)

4. Fletcher, H. A. et al. Human newborn bacille Calmette-Guerin vaccination and risk of tuberculosis disease: a case-control study. BMC medicine 14, 76, doi:10.1186/s12916-016-0617-3 (2016)

Supplementary Table 2: MSD assay panels

Cytokine/ Chemokines	TB disease Median con. pg/mL (interquartile range)	Household controls Median con. pg/mL (interquartile range)
**Pro-inflammatory panel**		
**IL-1β**	721.1 (471.6-1141.0)	412.9 (251.1-781.8)
**IL-4**	0.3 (0.2-0.6)	0.4 (0.3-0.7)
**IL-10**	2.4 (1.1-6.7)	2.2 (1.1-3.5)
**IL-12p70**	0.4 (0.1-0.8)	0.32 (0.1-0.8)
**IFN-γ**	1334.0 (433.2-3983.6)	949.4 (120.8-3443.3)
**TNF-α**	534.3 (256.5-1219.7)	275.1 (136.4-507.5)
**Cytokine panel**		
**GM-CSF**	7.9 (2.8-27.8)	13.9 (7.6-39.9)
**IL-15**	2.7 (2.1-3.6)	1.4 (1.2-1.7)
**IL-17A**	2.2 (1.5-3.8)	3.5 (2.1-7.8)
**IL-5**	1.0 (0.6-2.2)	2.3 (1.0-8.7)
**IL-7**	14.3 (10.9-20.1)	9.9 (7.5-11.7)
**VEGF**	68.4 (4.5-340.4)	55.8 (12.8-197.7)
**Chemokine panel**		
**Eotaxin-3**	452.5 (339.2-601.7)	516.5 (347.0-724.1)
**IL-8**	90120.5 (44280.3-163034.0)	52974.1 (31793.0-88650.3)
**IP-10**	30675.1 (8763.579453.4)	16810.8 (2698.5-48056.9)
**MCP-1**	25631.2 (13313.8-51421.9)	41255.9 (19811.1-70381.2)
**MDC**	1810.1 (1338.1-2392.9)	2549.9 (1920.3-3178.7)
**MIP-1β**	16520.6 (6813.2-24881.0)	11197.2 (7364.4-23343.1)
